# Zn/Cu Levels in the Field of Autism Disorders: A Systematic Review and Meta-analysis

**Published:** 2015

**Authors:** Fatemeh SAYEHMIRI, Nasim BABAKNEJAD, Somayeh BAHRAMI, Kourosh SAYEHMIRI, Mojtaba DARABI, Mostafa REZAEI-TAVIRANI

**Affiliations:** 1 Biochemistrist, Ilam University of Medical Sciences Research Committee, Ilam, Iran; 2 Department of Biostatistics, Department of Social Medicine, School of Medicine, University of Ilam, Ilam, Iran; 3 Proteomics Research Center, Shahid Beheshti University of Medical Sciences, Tehran, Iran

**Keywords:** Zinc, Cupper, Concentration, Autism spectrum disorders, Metaanalysis

## Abstract

**Objective:**

There is probably a relationship between zinc/cupper concentration in individuals with autism. The present review was written to estimate this probability by using meta-analysis method.

**Martials & Methods:**

In this meta-analysis of Fixed Effect Model, by searching PubMed, Scopus and Google scholar databases, 11 articles were selected and verified published in 1978 to 2012. I² statistics were calculated to examine heterogeneity. The information was analyzed by R and STATA Ver. 11.2.

**Results:**

Due to non-uniform measurement methods of Zn/Cu concentrations, the concentration of these elements was measured in various subgroups (plasma, hair and general) in both study cases and controls. There was a significant statistical difference between plasma OR=0.252 (95% CI: -0.001-0.504) and hair OR=0.27(95% CI: 0.059-0.481, P=0.01) concentrations of Zn/Cu statuses between controls and autistic patients. Using a Fixed Effects Model, the overall integration of data from the two groups was significant as risk factor OR=0.31(95% CI:0.16-0.46, P=0.001).

**Conclusion:**

Significant correlation existed between Zn/Cu levels and the development of autistic disorders in general analysis. Therefore, Zn/Cu levels could be mentioned as a pathogenesis reason of autism spectrum disorders.

## Introduction

Autism is a long-term disability characterized by social deficits, social imagination, language impairments, and repetitive behaviors (1). It is rare, but data indicates the prevalence of this disorder is 20/10,000 births (2). Autism is a neural development disorder with characteristics of impaired social interaction, verbal or non-verbal communication disorders, and repetitive restricted behavior. In most cases, the diagnostic criteria involve early diagnosis of symptoms before the child is three year old (3). This disease alters the connections and organization of synapses and nerve cells, which in turn alters information processing in the brain. Nonetheless, the way this phenomenon occurs is not yet fully understood (4). Despite having complex and vague genetics, autism has a strong genetic root, which can be described as scarce mutations or rare combinations of common variants of genes (5). In very few cases, agents, which cause birth defects, are considered responsible for autism (6). There are, however, controversies about other proposed environmental factors such as exposure to heavy metals, pesticides, or early vaccinations (7). The vaccine hypotheses are biologically implausible and lack convincing scientific evidence (8). The prevalence of autism is about 1–2/1,000 people worldwide, and occurs about four times more often in boys than girls (9). The environmental factors, which can prove important in future research, include particular foods, infectious diseases, heavy metals, solvents, diesel exhaust, PCBs, phthalates and phenols used in plastic products, pesticides, brominated flame retardants, alcohol, smoking, illicit drugs, vaccines(9) and prenatal stress (10). Zinc levels in plasma, hair, and nails in autistic patients have been measured which were not normal (11). The reported findings are not the same, so that in some cases zinc deficiency has been reported in individuals diagnosed with autistic spectrum disorders, but in other studies, autistic children had similar plasma zinc levels to neurotypical children (2, 11, 12). Plasma copper values were also determined on each blood specimen, and low plasma zinc was associated with an increased plasma copper level (13). Probably, there is a correlation between plasma, hair and teeth zinc and copper, and severity of symptoms associated with autism (14-17). Several studies have suggested a disturbance in the copper (Cu) and zinc (Zn) metabolism in ASDs (autism spectrum disorders) (6,8,14,16,18-27). Zinc deficiency, excess Cu levels, and low Zn/Cu ratio are common in children diagnosed with ASD. Due to the lack of uniform results and considering the impact of this element in symptoms and improvement of this disease, this meta-analysis study was conducted.

## Martials & Methods

**Study method**

**Fig 1:**
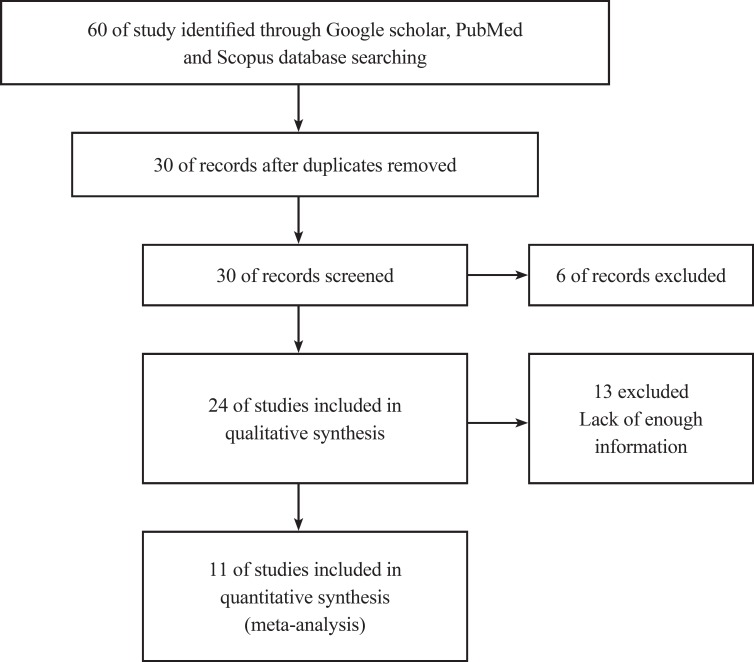
Study flowchart

A systematic review and Meta-analysis using PubMed, Google scholar and Scopus databases was undertaken to identify any study published in 1978-2012, in English, reporting Zn and Cu concentrations in individuals with autism. Databases were searched using the keywords ‘Autism Spectrum Disorders’, ‘autism’, ‘Zinc concentration’, ‘Copper concentration’, ‘Copper/Zinc concentration’, ’Cu/Zn concentration’, ‘trace element concentrations’ and their combinations. Eligible studies, including epidemiologic manuscripts, were analyzed for Zn and Cu levels in autistic patients by measuring the concentration of these elements in any of the following biological sample specimens: blood/ serum, nails, hair and teeth. All papers, with keywords presented in their titles or abstract ZZts, were used in the initial list and other unrelated articles were eliminated. Studies were excluded if they were not written in English; had insufficient data; if they were reviews; or if they were not epidemiologic studies. All the abstracts were reviewed and duplicates excluded. The following variables were extracted from each paper: sample characteristics (first author’s last name, year of publication, sample size, sample age, location), Zinc concentrations, Copper concentration, zinc to copper ratio, Mean difference, Zinc and Copper screening method and sample specimens (Figure 1).

**Statistical analysis**

Studies were combined based on the sample size, mean and standard deviation. The difference between the average variance of the normal distribution was calculated using the formula of two integrated variance. To assess heterogeneity of the studies, Cochran test and the I2 index were used. Due to significant heterogeneity in the studies, random effects model was used. To examine publication bias, Begg Plot and regressions method were used. P-value less than 5% was considered as a significant heterogeneity test. Sensitivity analyses were pre-specified. Statistical analyses were performed using STATA version 12.

## Results

**Fig 2:**
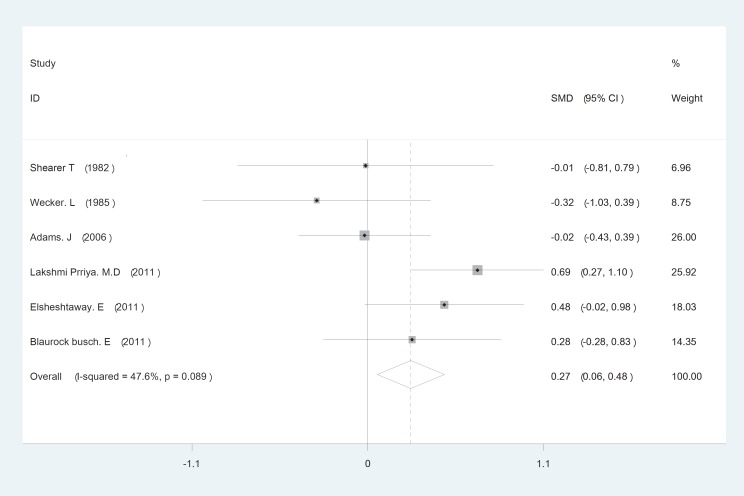
Forest plots for the hair and nail Zn/Cu statues difference between autistic patients and healthy individuals. The area of each square is proportional to the percentage weight of each individual study in the meta-analysis (CI 95%). In this chart studies are stored in order of year publication and author’s names, based on a fixed effects model

**Fig 3:**
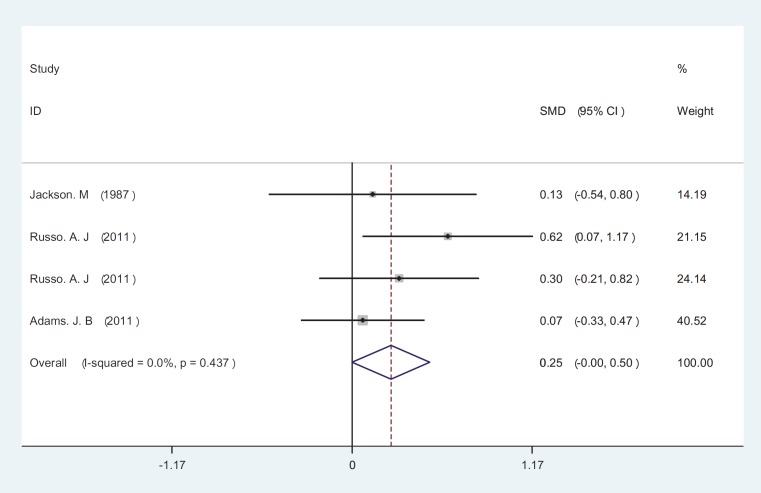
Forest plots for the plasma Zn/Cu statues difference between autistic patients and healthy individuals. The area of each square is proportional to the percentage weight of each individual study in the meta-analysis (CI 95%). In this chart studies are stored in order of year publication and author’s names, based on a fixed effects model

**Fig 4:**
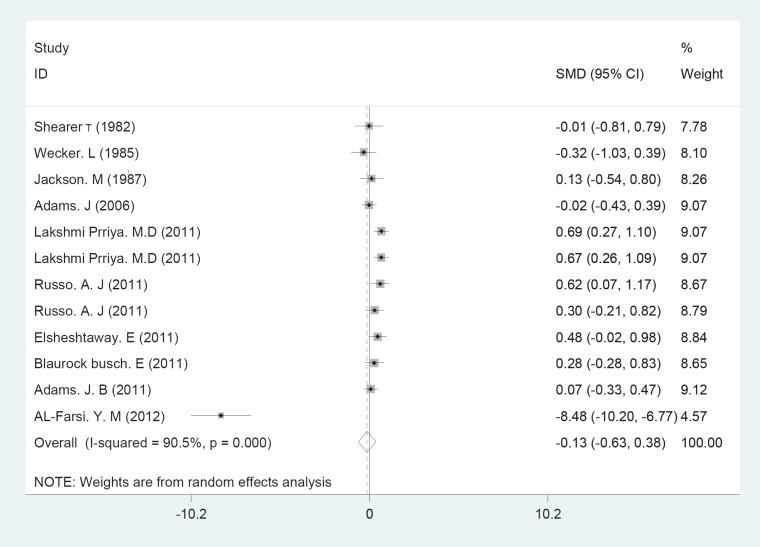
Forest plots for the studies Zn/Cu statuses combination difference between autistic patients and healthy individuals. The area of each square is proportional to the percentage weight of each individual study in the meta-analysis (CI 95%). In this chart studies are stored in order of year publication and author’s names, based on a random effects model

**Fig 5:**
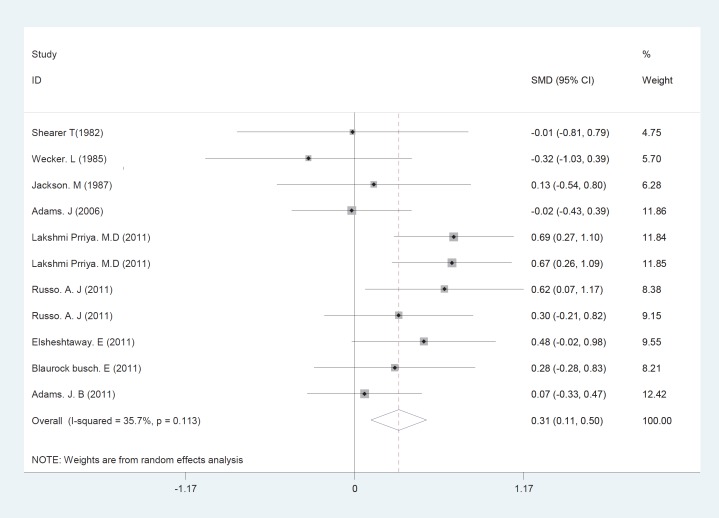
Sensitivity analyses forest plots for the studies Zn/Cu statuses combination difference between autistic patients and healthy individuals. The area of each square is proportional to the percentage weight of each individual study in the meta-analysis (CI 95%). In this chart, studies are stored in order of year publication and author’s names, based on a fixed effects model

**Fig 6.**
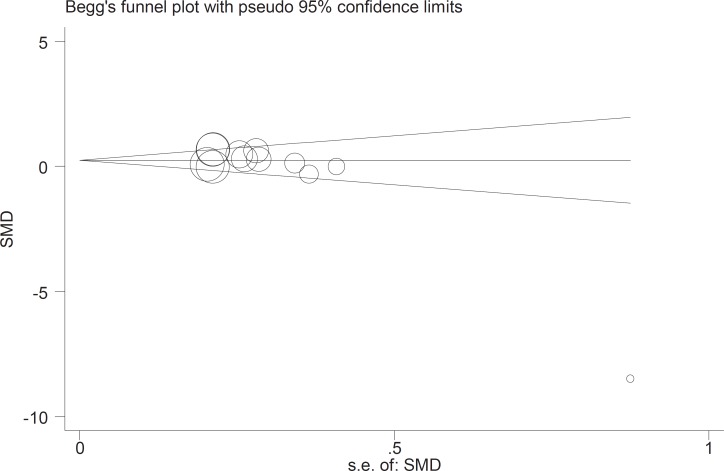
Begg’s funnel plot for publication bias in the risk difference (RD) analysis. The diameter of each circle represents the weight in the metaanalysis

The initial search returned 60 citations. Of these, 29 studies were discarded after reviewing the abstracts while the full text of the remaining 21 citations was examined in more detail; among those, 11 were appropriate for inclusion in the meta-analysis. The standard unit for measuring Zn and Cu concentrations in many articles was microgram per gram (µg/gr). However, all studies 1). Zn/Cu concentrations were measured in plasma, hair and total. Due to non-uniform measurement methods of Zn and Cu concentration, the levels of these elements were measured in various subgroups in both cases and controls. There was significance statistical difference between plasma OR=0.252 (95% CI: -0.001-0.504); hair and nail OR=0.27(95% CI: 0.059-0.481, P=0.01) and Zn/Cu statuses analysis between controls and autistic patients after sensitivity analysis and removal of AlFarsi’s study. The overall integration of data from the two groups showed there was no significance statistical difference between Zn/Cu concentrations and between autistic patients and healthy controls -0.129 (95% CI: -0.63 – 0.37). Using a Random Effects model, the overall integration of data from the two groups revealed a significant difference between Zn/Cu status. After deleting Al-Farsi studies (sensitivity analysis) mean difference was estimated 0.31 (95% CI: 0.16 – 0.46; P=0.000). Heterogeneity was not significant, so we used Fixed Effects model to combine studies. Figures 2-4 show the results of meta-analysis for each study and for studies combination based on fixed and also fixed and random effects model, respectively. Additionally, sensitivity analyses by running metaanalysis using just the higher quality studies were conducted, excluding the Al-Farsi et al. (Figure 5). These charts are given based on years of research and the author’s name. Publication bias was detected by drawing Beggs funnel plot in the meta-analysis. This diagram shows that there is no significant publication bias (P=0.00); this means, both of the tests (positive and negative results) have been published (Figure 6).

**Table 1:** Study characteristics

**References**	**Year** **of publication**	**Country** **City**	**Mean age**		**Case**	**Control**	**Matrix**	**95% CI**		**Mean difference**	**type of Zinc and Copper measurement**
			**Case**	**Control**							
								**Lower**	**upper**		
23	1978	London	7-16	7-17	20	30	plasma	-1.162	-0.007	-0.584	Atomic Absorption Spectroscopy
26	1985	USA	2-11	2-11	12	21	Hair	-1.408	0.050	-0.679	Atomic Absorption Spectroscopy
25	1982	USA	8	8	12	12	Hair	-0.841	0.786	-0.014	Atomic Absorption Spectroscopy
28	2006	USA	3-6	3-6	51	40	Hair	-0.149	0.683	0.267	Inductively Coupled Plasma–Mass spectrometry (ICP-MS)
29	2011	India	4-12	4-12	45	50	Hair	-1.229	-0.390	-0.810	Atomic Absorption Spectroscopy
29	2011	India	4-12	4-12	45	50	Nail	-1.189	-0.354	-0.772	Atomic Absorption Spectroscopy
16	2011	USA	38	42	73	16	Plasma	-0.831	0.254	-0.288	ICP-MS
16	2011	USA	11.7 ± 5.62	11.7 ± 5.62	79	18	Plasma	-0.801	0.226	-0.288	ICP-MS
30	2011	Egypt	4.1 ± 0.8	4.1 ± 0.8	32	32	Hair	-3.802	-2.343	-0.073	Atomic Absorption Spectrophotometer
31	2011	Egypt	5.29±1.9	6.25 ± 2.39	25	25	Hair	-1.464	-0.300	-0.882	ICP-MS
14	2011	USA	5.16	5.16	55	44	Plasma	-0.543	0.340	-0.057	ICP-MS
19	2012	Oman	3_14	3_14	27	27	Hair	0.899	2.113	1.506	ICP-MS

## Discussion

In this meta-analysis, the plasma, hair and nail status of Zn/Cu were examined and showed that there was significance statistical difference between plasma Zn/Cu concentrations in autistic patients and healthy controls (0.25 (95% CI: 0.00 - 0.504)) by using a random effects model. However, the error is significant at 0.06 (P=0.051). Zn/Cu mean levels in healthy subjects were higher than autistic individuals were. The results obtained from the hair and nail has clarified that there was no significant difference of Zn/Cu concentrations between autistics and healthy subjects (P=0.13). In all the twelve studied analysis, which applied individual levels of Zn/Cu measured in plasma and hair, there was no significant difference in Zn/Cu levels between autistic patients and healthy individuals OR=-0.12(95% CI:-0.63 - 0.37, P=0.61). After deleting Al-farsi studies there was significant association between Zn/Cu and autism (OR=0.31(95% CI:0.16 - 0.46, P=0.000). Nonetheless, these differences were often weak and some studies showed positive results while others showed negative results (16-31). Low Zn/Cu ratio is common in children diagnosed with ASD (21). Zinc is in a balance with Cu in the blood, and changes in these two trace elements are in an inverse relationship. This can, in large scale, be explained because of cytokine regulation of the metabolism of the two elements, with the same cytokines causing enhancement of the cellular uptake of Zn and enhancement of the production of ceruloplasmin in the liver. A low plasma Zn concentration is nearly always associated with a high serum Cu concentration. The normal Zn to Cu ratio in children and adults is close to 1:1, besides, the plasma Zn/serum Cu ratio may be used as a rapid method of determining the functional state of the metallothionein system (18, 19). Low Zn/Cu ratios can be associated with total body Zn deficiency or accumulation of toxic metals, which can act as Zn-antagonists. Hg toxicity may be a major cause of MT dysfunction in children diagnosed with ASD, which may be reflected in the Zn/Cu ratio (19, 20). The toxic metals Hg and Cd, similar to that proposed above for oxidative stress due to genetic disturbances, might have opposite net effects on Zn and Cu metabolism, as enhanced MT induction in the liver might affect Cu excretion via the bile more than it affects the mobilization of this element from the liver to the blood, while for Zn it is the rate of mobilization to the blood which is more strongly affected (21). The frequency of zinc deficiency, copper toxicity and low Zn/Cu ratio in children with autism spectrum disorders may indicate decrement in metallothionein system functioning attributed to MTs including the sequestration and dispersal of metal ions, primarily in zinc and copper homeostasis specifically in regulation of the biosynthesis and activity of zinc metalloproteins, most notably, zinc-dependent transcription factors. A retrospective review of plasma zinc, serum copper and Zn/Cu was performed. The entire cohort’s mean zinc level was 77.2 microg dl (-1), mean copper level was 131.5 microg dl(-1), and mean Zn/Cu was 0.608, which was below the 0.7 cut-off of the lowest 2.5% of healthy children. The plasma zinc/serum copper ratio may be a biomarker of heavy metal, particularly mercury, toxicity in children with ASDs (22, 23). Medical nutrition therapy and use of dietary supplements is a suggestion for curing this disease (26). However, many risk factors are included in the etiology of autism. One of them is essential elements deficiency. Therefore, it is important to determine the trace elements concentrations in humans to monitor and assess their impact on health (24, 25). The major limitation of this study is the conduct of a meta-analysis in the presence of high heterogeneity among the studies. Fixed effects model was used to try to mitigate the heterogeneity as an issue, and sensitivity analyses changed the results. Other limitations and weaknesses were commonly related to the methodology of reviewed studies. Some of these weaknesses are as follows: 1) Lack of a same method of measurement for the variances. 2) Selection of cases from women referred to the health centers compared to a random selection. 3) Lack of information about nutrition and lifestyle of participants. 4) Various kinds of screening methods and lack of same standard unit for measuring selenium concentrations in different articles. In conclusion, this meta-analysis, which was based on fixed effect model, indicated that in the etiology of autism, significant correlation existed between Zn/ Cu levels based on sensitivity analysis by excluding al-Farsi et al. (19). Zn/Cu supplements can be used in randomized clinical trials for the nutritional therapy of autistic patients.
